# Education and cardiovascular diseases: a Mendelian randomization study

**DOI:** 10.3389/fcvm.2024.1320205

**Published:** 2024-02-15

**Authors:** Wei Liu, Quan Lin, Zongjing Fan, Jie Cui, Yang Wu

**Affiliations:** Department of Cardiology, Dongfang Hospital, Beijing University of Chinese Medicine, Beijing, China

**Keywords:** education, years of schooling, cardiovascular diseases, Mendelian randomization, causality

## Abstract

**Background:**

Observational studies have indicated a potential association between education and cardiovascular diseases (CVDs). However, uncertainties regarding the causal relationship persist. Therefore, this study aimed to investigate whether higher levels of education causally reduce the risks of CVDs.

**Methods:**

Employing a two-sample Mendelian randomization (MR) design, our study examined the relationship between education and ten different CVDs. Utilizing data from the IEU Open GWAS database, relevant single nucleotide polymorphisms (SNPs) were identified through stringent screening criteria. Causality was assessed using the inverse-variance weighted (IVW), ME-Egger regression, and weighted median methods. Sensitivity analyses, including heterogeneity and pleiotropy tests, were conducted to ensure the robustness of our findings.

**Results:**

Our study identified a genetic predisposition associated with an additional 3.6 years of education, which significantly reduced the risk of various CVDs. Specifically, this genetic factor was found to lower the risk of type 2 diabetes by 46.5%, coronary heart disease by 37.5%, ischemic stroke by 35.4%, cardiac-related mortality by 28.6%, heart failure by 28.2%, transient ischemic attack by 24%, atrial fibrillation by 15.2%, peripheral artery disease by 0.3%, and hypertension by 0.3%. However, no significant evidence revealed a causal relationship between education and pulmonary embolism.

**Conclusion:**

Our study provides robust evidence supporting the role of higher educational attainment in reducing the incidence of various cardiovascular diseases, including type 2 diabetes, coronary heart disease, ischemic stroke, cardiac-related mortality, heart failure, transient ischemic attack, atrial fibrillation, peripheral artery disease, and hypertension. However, the impact of education on pulmonary embolism remains inconclusive.

## 1 Introduction

Cardiovascular diseases (CVDs) encompass a wide range of conditions that have a significant impact on quality of life and contribute to global mortality (1, 2). Ischemic heart disease and stroke are major causes of disability among individuals aged 50 and above (3). The prevalence of CVDs has been steadily increasing worldwide for several decades (4). Various factors, including cardiometabolic, environmental, and social risks, contribute to the development of CVDs. While causal relationships have been established for certain risk factors like hypertension and diabetes, the impact of other factors, such as education, on the causality of CVDs remains uncertain. Further research is warranted to comprehensively understand these complex interactions.

Those with higher levels of education tend to exhibit better health, experience fewer illnesses, and have longer lifespans than those with lower levels of education (5, 6). Research has demonstrated the significant impact of socioeconomic risk factors, including education, on the development of CVDs (7–10). People with lower levels of education generally have worse cardiovascular health, more comorbidities, and a higher overall risk for developing cardiovascular disease (8). However, it is important to note that while traditional observational studies have established an association between education and CVDs, they do not prove causality due to various limitations. To gain deeper insights into the causes of CVDs and develop effective preventive measures, it is crucial to investigate the potential causal relationship between education and CVDs. Although randomized controlled trials (RCTs) would be the ideal approach, practical challenges such as the long latency period between exposure and outcomes, as well as ethical concerns related to limiting children's education, render such trials unfeasible. Therefore, alternative research methods should be implemented to effectively evaluate causality. Improving our understanding in this area will aid in developing targeted interventions and strategies to alleviate the burden of CVDs across different educational backgrounds.

Mendelian randomization (MR) research utilizes single nucleotide polymorphisms (SNPs) as instrumental variables (IVs) to establish models and infer causal effects (11). By randomly assigning exposed IVs during conception, unobserved confounders can be eliminated, allowing for the assessment of causal relationships between exposure and disease (12). Recent advances in genome-wide association studies (GWAS) have yielded abundant data on genetic variants and their associations with phenotypes. Of which the IEU Open GWAS database is a manually curated collection of complete GWAS summary datasets. It is developed at the MRC Integrative Epidemiology Unit (IEU) at the University of Bristol and made available as open source files for download or by querying a database of the complete data. Leveraging this data, MR studies enable the evaluation of the impact of risk factors on outcomes without requiring additional patient recruitment. MR studies have been widely employed in various diseases for causal inference. In this particular study, two-sample MR analyses were conducted to investigate the potential causal association between education and ten cardiovascular diseases. The objective of this research is to provide valuable insights that can contribute to the prevention and treatment of CVDs.

## 2 Methods

### 2.1 Study design

**Figure 1:**
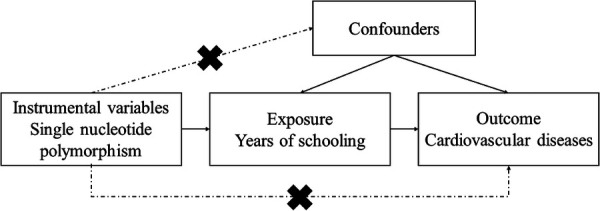
Description of the study design: three core hypotheses of the Mendelian randomization study.

In our study, we conducted multiple MR analyses to investigate the relationship between education and CVDs (11). We comprehensively analyzed various types of CVDs, including coronary heart disease, hypertension, atrial fibrillation, cardiac death, pulmonary embolism, transient ischemic attack, peripheral artery disease, heart failure, type 2 diabetes, and ischemic stroke. By utilizing data from GWAS, we obtained valuable insights into the genetic basis of these diseases. We employed the two-sample MR method to explore potential causal effects between education and the development of CVDs (Figure 1). It is important to note that MR studies rely on three fundamental assumptions: establishing a strong association between genetic variation and the exposure variable, ensuring no association between the genetic variation and potential confounding factors, and confirming no association between genetic variation (apart from its effect on the exposure variable) and the outcome of interest (13). Furthermore, our study exclusively utilized publicly available summary data, which precluded the necessity for ethical approval.

### 2.2 Data sources

The analysis conducted in our study utilized summary-level data obtained from GWAS, which were predominantly conducted on individuals of European ancestry. To explore the genetic variants associated with education, we relied upon a large-scale genetic association analysis published in Nature Genetics in 2018. This analysis included approximately 1.1 million individuals and analyzed a vast dataset of 10,101,242 SNPs, reported educational attainment in people of European descent (14). We defined educational attainment in the same manner as that in this study in which educational attainment was defined by whether the participant attained a given level of schooling based on the International Standard Classification of Education 1997 classification scale. The results provided an estimate of relative risk caused by each standard deviation, which is 3.6 years, increase in the years of schooling. For coronary heart disease, our study included 60,801 cases and 123,504 controls of European ancestry, with 9,455,779 SNPs analyzed. In the investigation of hypertension, we analyzed 1,237 cases and 359,957 controls of European ancestry, examining 9,646,741 SNPs. More information about the studies on other cardiovascular diseases can be found in Table 1. Notably, all the analysis data were aggregated and accessible through the IEU Open GWAS database (https://gwas.mrcieu.ac.uk/).

**Table 1:** Details of studies included in Mendelian randomization analyses.

Trait	GWAS ID	Years	Sample size	Number of SNPs
Years of schooling	ieu-a-1239	2018	766,345	10,101,242
Coronary heart disease	ieu-a-7	2015	184,305	9,455,779
Hypertension	ukb-d-I9_HYPTENS	2018	361,194	9,646,741
Atrial fibrillation	ebi-a-GCST006414	2018	1,030,836	33,519,037
Death due to cardiac causes	ukb-d-I9_K_CARDIAC	2018	361,194	10,071,648
Pulmonary embolism	finn-b-I9_PULMEMB	2021	218,413	16,380,466
Transient ischemic attack	finn-b-G6_TIA	2021	214,634	16,380,437
Peripheral artery disease	finn-b-I9_PAD	2021	213,639	16,380,453
Heart failure	ebi-a-GCST009541	2020	977,323	7,773,021
Type 2 diabetes	ebi-a-GCST006867	2018	655,666	5,030,727
Ischemic stroke	ebi-a-GCST006908	2018	440,328	8,296,492

### 2.3 Selection and validation of SNPs

In the selection process of suitable SNPs, we employed three key criteria. Firstly, SNPs were chosen based on the significant threshold of genome-wide association (*P* < 5 × 10^−8^) (15). Secondly, we conducted pairwise-linkage disequilibrium analysis to evaluate the independence among selected SNPs. SNPs with a correlation coefficient (*r*^2^) greater than 0.001, within a clumping window of 10,000 kb, were removed if they exhibited a higher *P*-value or were correlated with a larger number of SNPs (16, 17). Lastly, we validated the strength of individual SNPs using the F-statistic. SNPs with an F-statistic exceeding ten were considered powerful enough to mitigate potential bias. Prior to conducting the Mendelian Randomization (MR) analysis, we implemented data-harmonization steps to ensure that the effects of an SNP on exposure and outcome corresponded to the same allele.

### 2.4 MR analysis

In the analyzed study, we conducted an inverse-variance weighted (IVW) meta-analysis as the primary analysis method, employing a random-effects model (18). Additionally, we performed two sensitivity analyses using different methods. The first sensitivity analysis involved the weighted median method, which provides valid estimates if at least 50% of the information came from valid instrumental variables (19, 20). The second sensitivity analysis employed the MR-Egger method to evaluate the presence of horizontal pleiotropy in the selected instrumental variables (21, 22). To assess heterogeneity among the instrumental variables, we used Cochran's Q-value. Furthermore, we performed a leave-one-out sensitivity analysis to examine the potential impact of individual single nucleotide polymorphisms. The analytical procedures were carried out using R (version 4.3.1) and R Studio (version 2022.06.1 + 524). We considered statistical significance to be defined as *P* < 0.05.

## 3 Results

The included studies, published between 2015 and 2021, primarily focused on the European population. We identified 317 IVs that reached genome-wide significance levels, with all of them having F-statistics greater than ten.

**Figure 2:**
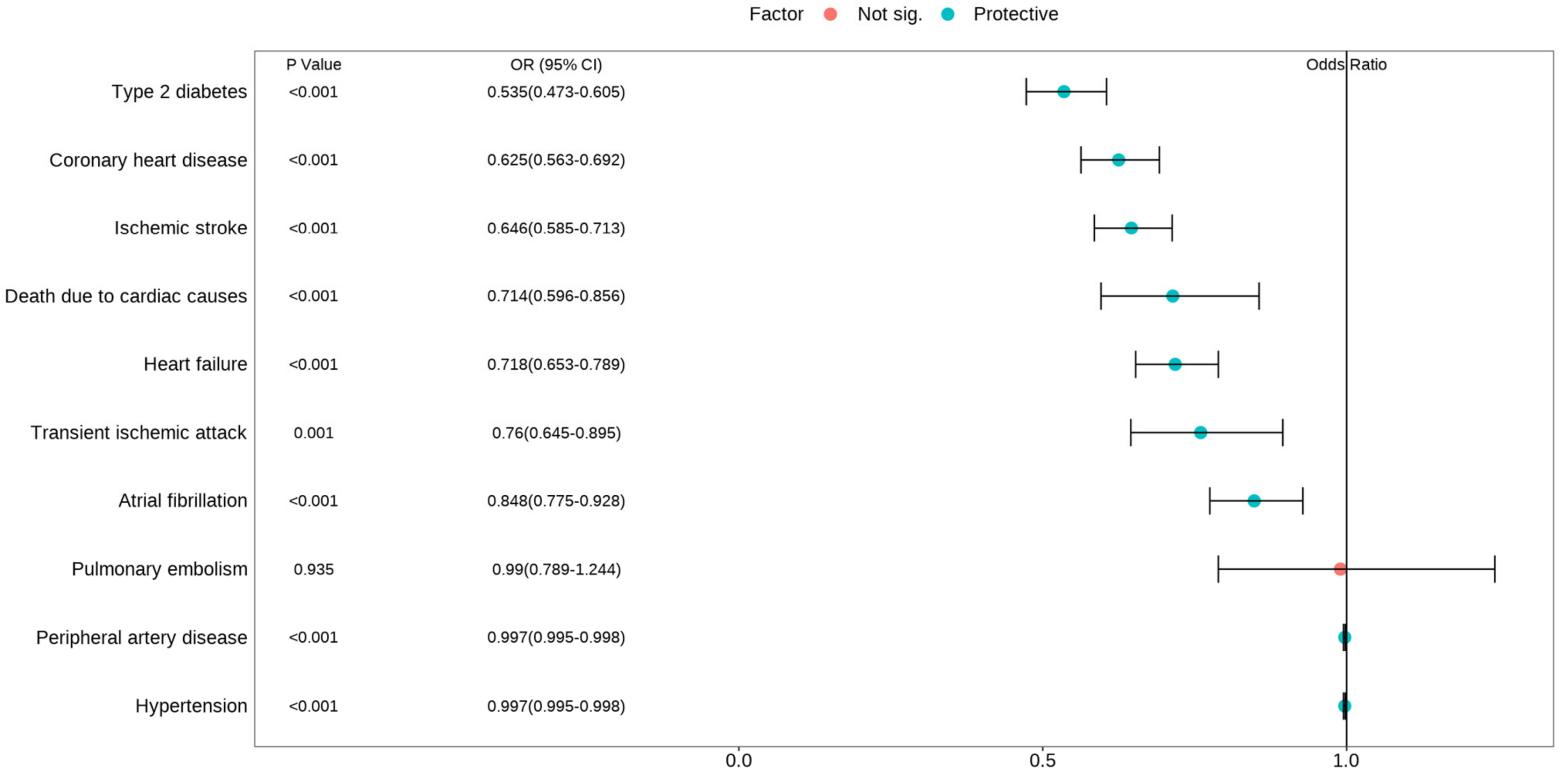
Associations of genetically predicted education with cardiovascular diseases.

The IVW analysis demonstrated a significant inverse association between higher educational attainment and nine cardiovascular diseases (Figure 2). These diseases, ranked by decreasing magnitude of association, included type 2 diabetes (odds ratio (OR) = 0.535; 95% confidence interval (CI), 0.473–0.605; *P* < 0.001), coronary heart disease (OR = 0.625; 95% CI, 0.563–0.692; *P* < 0.001), ischemic stroke (OR = 0.646; 95% CI, 0.585–0.713; *P* < 0.001), death due to cardiac causes (OR = 0.714; 95% CI, 0.596–0.856; *P* < 0.001), heart failure (OR = 0.718; 95% CI, 0.653–0.789; *P* < 0.001), transient ischemic attack (OR = 0.760; 95% CI, 0.645–0.895; *P* = 0.001), atrial fibrillation (OR = 0.848; 95% CI, 0.775–0.928; *P* < 0.001), peripheral artery disease (OR = 0.997; 95% CI, 0.995–0.998; *P* < 0.001), and hypertension (OR = 0.997; 95% CI, 0.995–0.998; *P* < 0.001). However, there was no evidence to suggest a potential causal relationship between education and pulmonary embolism (OR = 0.990; 95% CI, 0.789–1.244; *P* = 0.935). Most cardiovascular diseases showed consistent estimates but with low precision in the weighted-median and MR-Egger analyses (Table 2). No directional pleiotropy was detected, although heterogeneity was observed to be higher in certain CVDs. Therefore, an IVW meta-analysis under a random-effects model was employed to mitigate the influence of heterogeneity.

**Table 2:** Mendelian randomization estimates of the associations between education attainment and cardiovascular diseases.

Outcome	IVW method		MR-Egger		Weighted median method		Pleiotropy		Heterogeneity	
	OR (95% CI)	*P*	OR (95% CI)	*P*	OR (95% CI)	*P*	Intercept	*P*	Q	*P*
Coronary heart disease	0.625 (0.563–0.692)	<0.001	0.753 (0.496–1.142)	0.183	0.643 (0.560–0.738)	<0.001	−3.00 × 10^−3^	0.365	393	<0.001
Hypertension	0.997 (0.995–0.998)	<0.001	1.002 (0.996–1.007)	0.582	0.996 (0.994–0.998)	<0.001	−6.59 × 10^−5^	0.085	362	0.016
Atrial fibrillation	0.848 (0.775–0.928)	<0.001	0.837 (0.586–1.195)	0.327	0.836 (0.754–0.927)	<0.001	1.89 × 10^−4^	0.939	616	<0.001
Death due to cardiac causes	0.714 (0.596–0.856)	<0.001	0.630 (0.312–1.271)	0.198	0.691 (0.534–0.894)	0.005	1.78 × 10^−3^	0.716	298	0.420
Pulmonary embolism	0.990 (0.789–1.244)	0.935	0.650 (0.269–1.571)	0.340	0.988 (0.704–1.386)	0.945	5.94 × 10^−3^	0.334	315	0.191
Transient ischemic attack	0.760 (0.645–0.895)	0.001	1.104 (0.585–2.086)	0.760	0.862 (0.679–1.095)	0.224	−5.27 × 10^−3^	0.234	325	0.104
Peripheral artery disease	0.997 (0.995–0.998)	<0.001	0.997 (0.992–1.003)	0.322	0.997 (0.995–0.999)	0.002	−4.58 × 10^−6^	0.905	366	0.010
Heart failure	0.718 (0.653–0.789)	<0.001	0.852 (0.584–1.242)	0.405	0.764 (0.676–0.863)	<0.001	−2.40 × 10^−3^	0.358	366	<0.001
Type 2 diabetes	0.535 (0.473–0.605)	<0.001	0.873 (0.527–1.447)	0.599	0.576 (0.504–0.658)	<0.001	−6.81 × 10^−3^	0.051	590	<0.001
Ischemic stroke	0.646 (0.585–0.713)	<0.001	0.684 (0.461–1.016)	0.061	0.627 (0.543–0.724)	<0.001	−8.08 × 10^−4^	0.768	329	0.160

**Figure 3:**
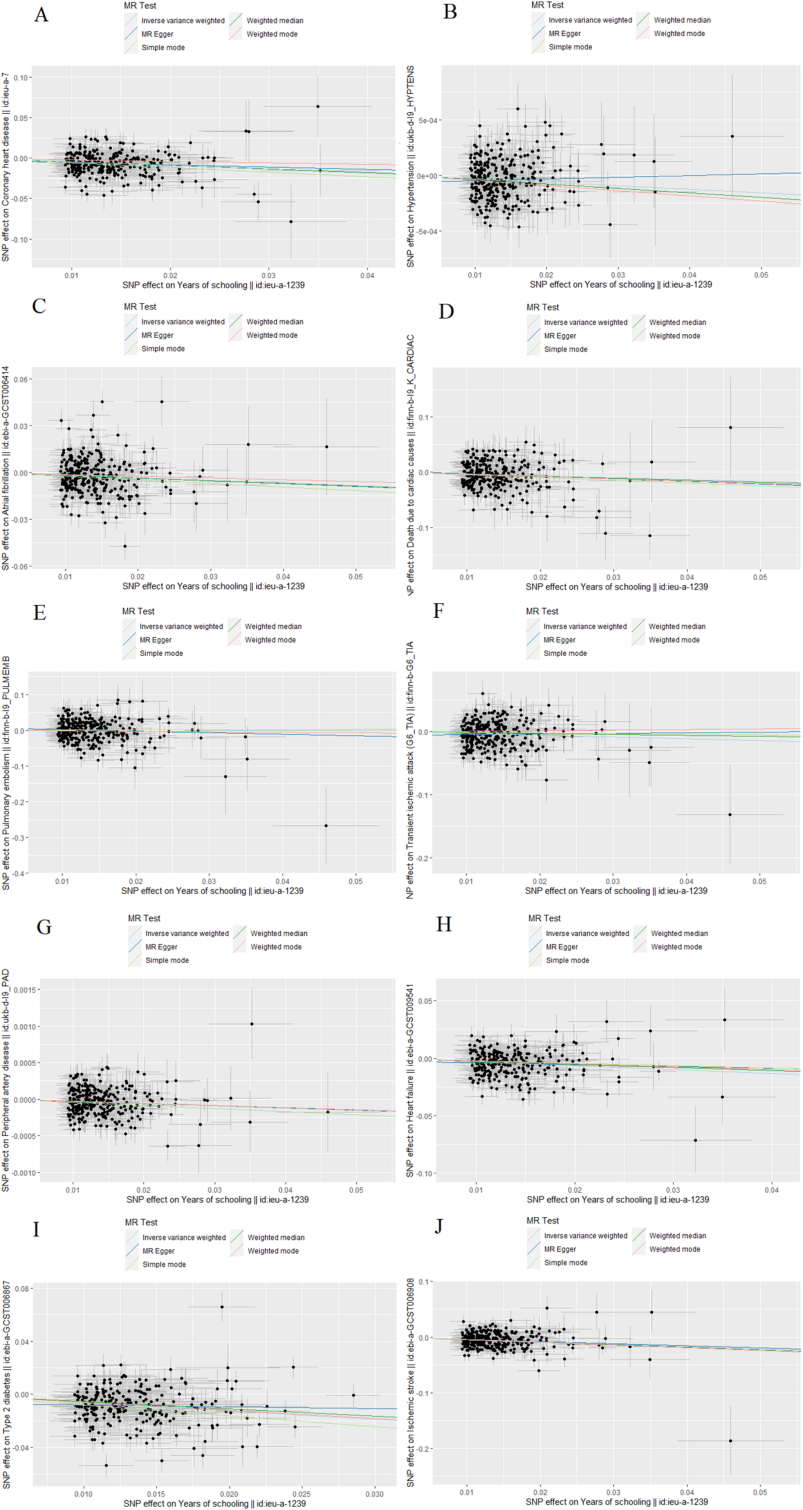
The scatter plot of Mendelian randomization analyses. (**A**) Coronary heart disease; (**B**) Hypertension; (**C**) Atrial fibrillation; (**D**) Death due to cardiac causes; (**E**) Pulmonary embolism; (**F**) Transient ischemic attack; (**G**) Peripheral artery disease; (**H**) Heart failure; (**I**) Type 2 diabetes; (**J**) Ischemic stroke.

**Figure 4:**
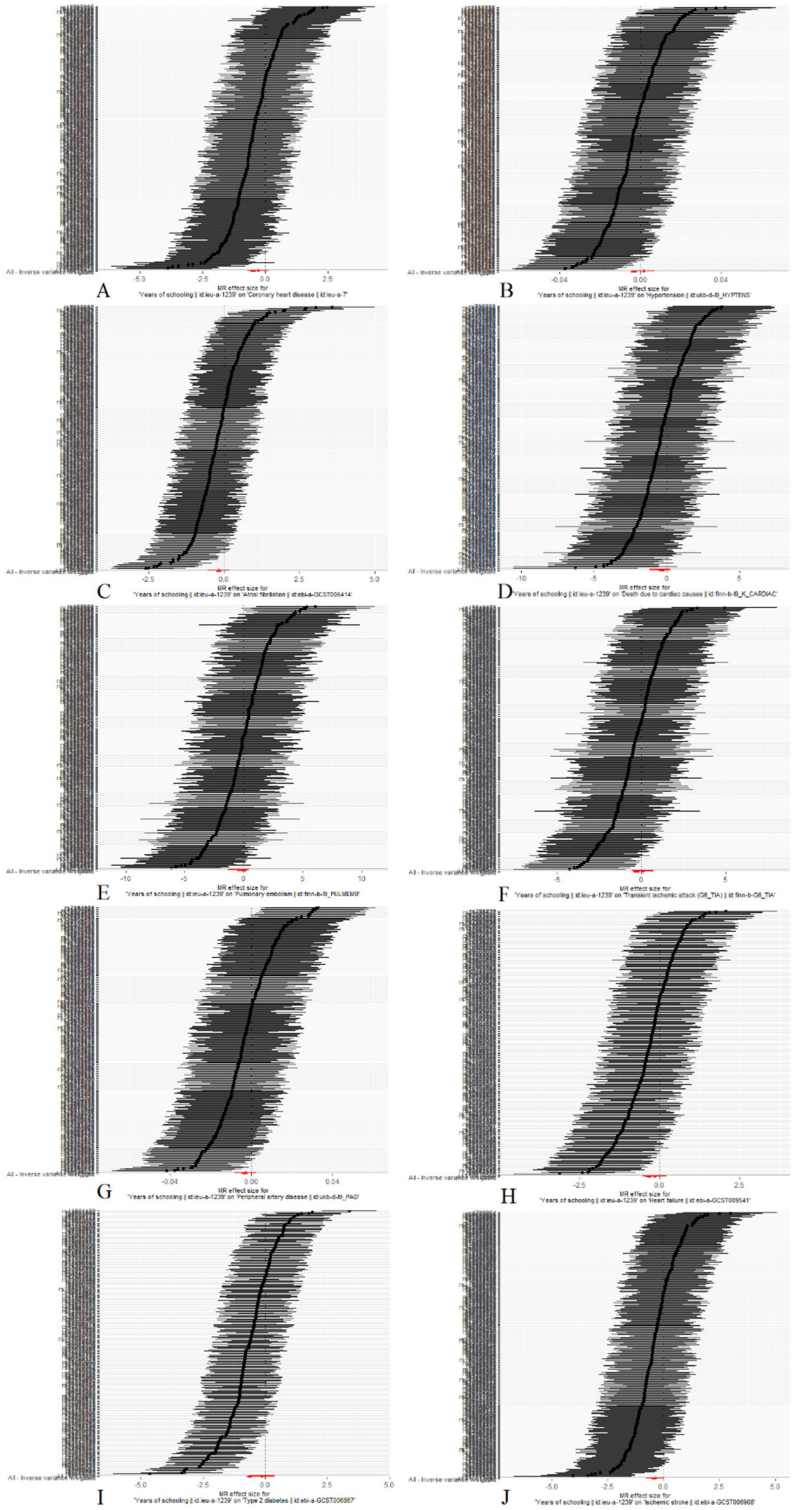
The forest plot of Mendelian randomization analyses. (**A**) Coronary heart disease; (**B**) Hypertension; (**C**) Atrial fibrillation; (**D**) Death due to cardiac causes; (**E**) Pulmonary embolism; (**F**) Transient ischemic attack; (**G**) Peripheral artery disease; (**H**) Heart failure; (**I**) Type 2 diabetes; (**J**) Ischemic stroke.

**Figure 5:**
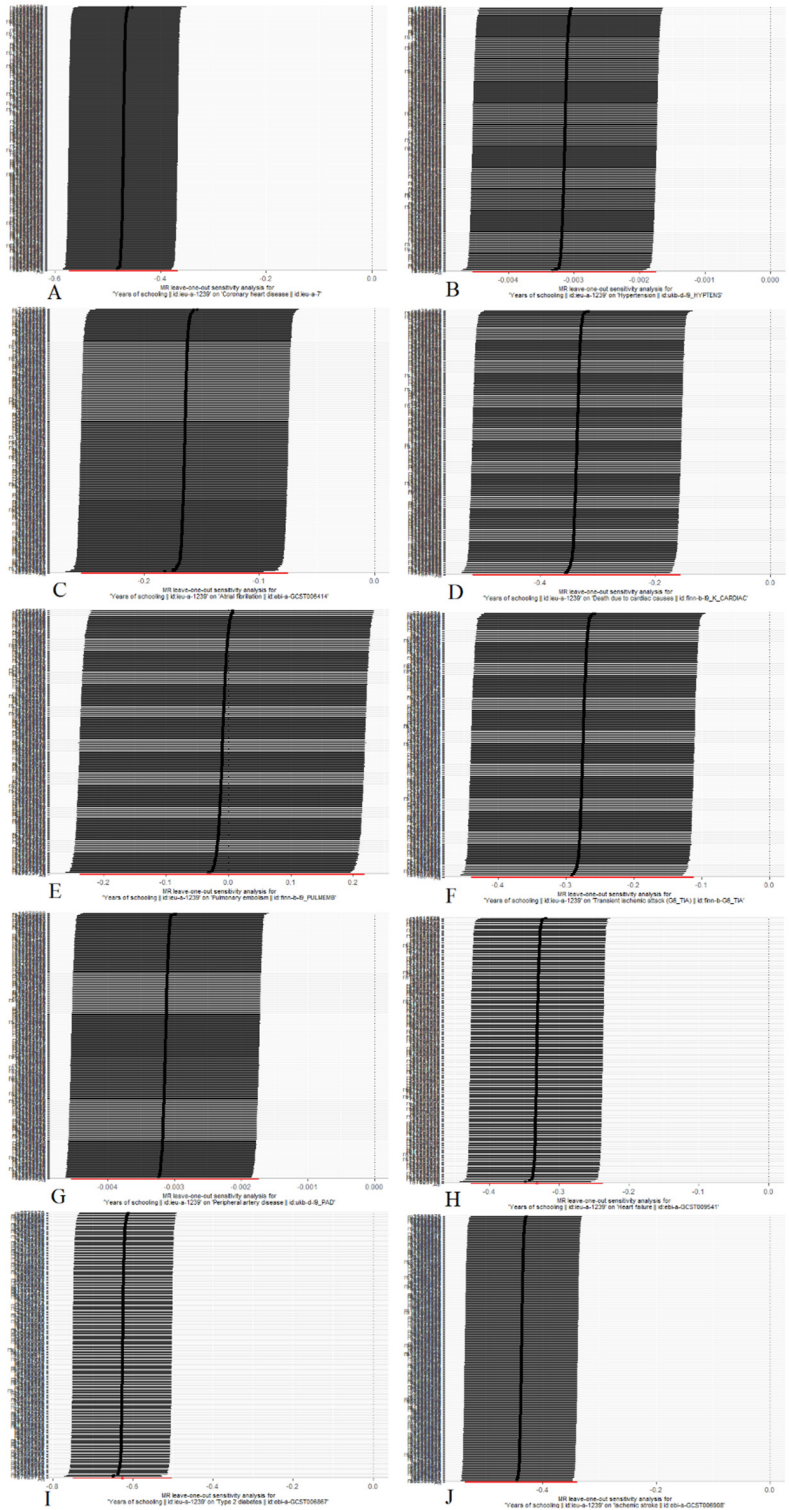
Mendelian randomization leave-one-out analyses. (**A**) Coronary heart disease; (**B**) Hypertension; (**C**) Atrial fibrillation; (**D**) Death due to cardiac causes; (**E**) Pulmonary embolism; (**F**) Transient ischemic attack; (**G**) Peripheral artery disease; (**H**) Heart failure; (**I**) Type 2 diabetes; (**J**) Ischemic stroke.

**Figure 6:**
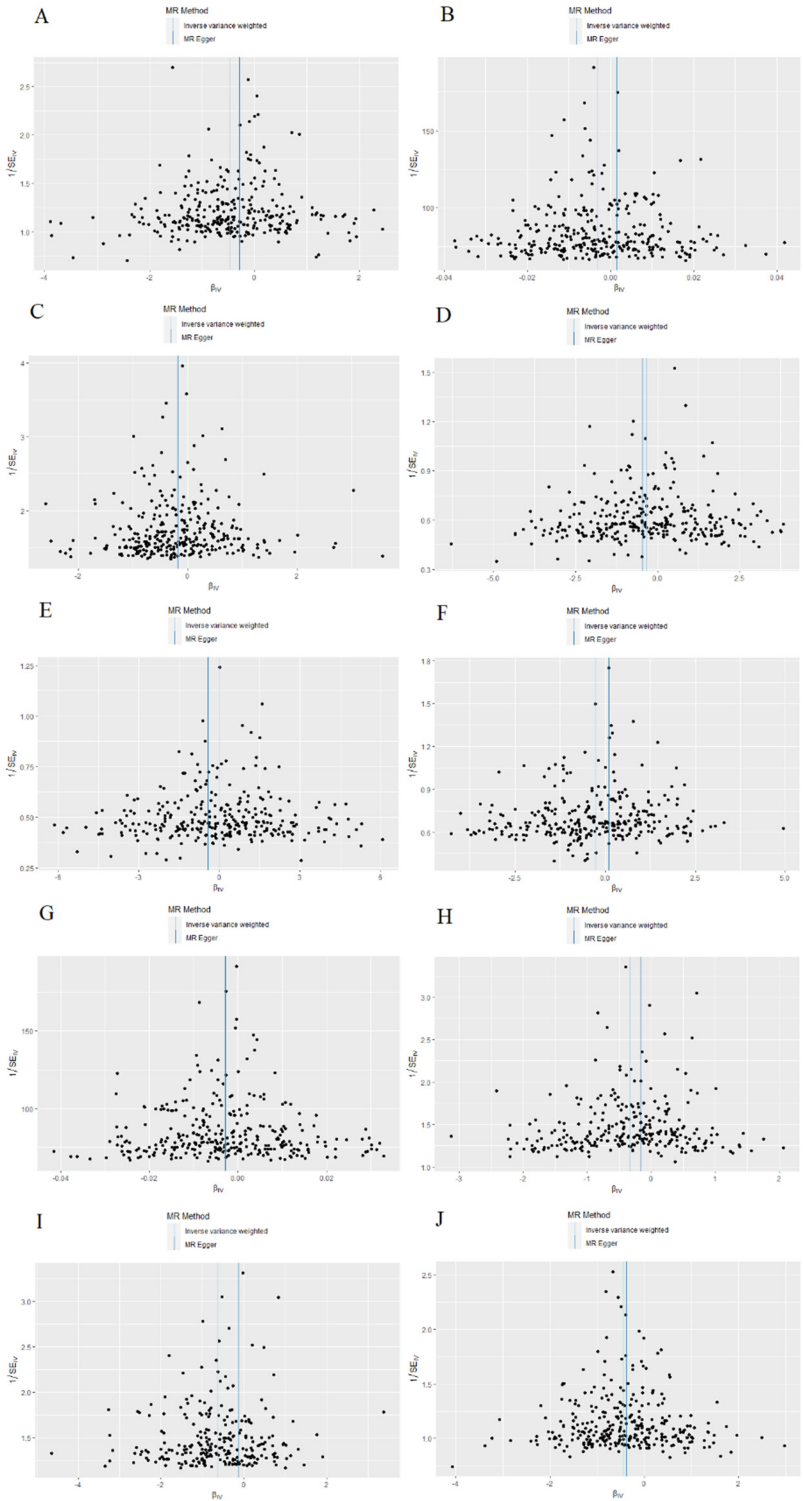
The funnel plot of Mendelian randomization analyses. (**A**) Coronary heart disease; (**B**) Hypertension; (**C**) Atrial fibrillation; (**D**) Death due to cardiac causes; (**E**) Pulmonary embolism; (**F**) Transient ischemic attack; (**G**) Peripheral artery disease; (**H**) Heart failure; (**I**) Type 2 diabetes; (**J**) Ischemic stroke.

Scatterplots depicting the relationship between education and CVDs can be found in Figure 3, while the forest plots displaying the association are provided in Figure 4. The leave-one-out sensitivity analyses, presented in Figure 5, indicate that the overall estimates were not significantly affected by any individual SNP. Additionally, the funnel plot in Figure 6 demonstrates no signs of horizontal pleiotropy.

## 4 Discussion

In our comprehensive two-sample MR analysis, we thoroughly examined the causal relationship between education and CVDs. Our findings demonstrated a significant association between higher educational attainment and a decreased likelihood of developing type 2 diabetes, coronary heart disease, ischemic stroke, cardiac-related mortality, heart failure, transient ischemic attack, atrial fibrillation, peripheral artery disease, and hypertension. Specifically, each additional 3.6 years of education resulted in a risk reduction of 46.5%, 37.5%, 35.4%, 28.6%, 28.2%, 24%, 15.2%, 0.3%, and 0.3% for the aforementioned CVDs, respectively. While education did impact peripheral artery disease and hypertension, the effect sizes were minimal. However, no statistically significant effect of education on pulmonary embolism was observed.

Education plays a pivotal role in determining health outcomes, particularly in the realm of cardiovascular diseases. A study encompassing 1.2 million individuals in Sweden from 1943 to 2007 discovered that compulsory education was associated with reduced mortality rates (6). Similarly, an investigation utilizing the UK Biobank dataset revealed that prolonged schooling mitigated the risk of diabetes and mortality (7). Moreover, consistent evidence suggests that higher educational attainment is correlated with a decreased lifetime risk of cardiovascular diseases (23). Regarding specific cardiovascular conditions, research demonstrated an inverse relationship between educational attainment and the likelihood of heart failure in patients with coronary heart disease (24). This highlights the potential benefit of implementing preventive measures for individuals with lower education levels to address survival disparities. Furthermore, a separate analysis uncovered an independent association between educational attainment and overall cardiovascular risk, underscoring the significance of education in maintaining cardiovascular health (8). Importantly, education emerged as the most reliable predictor of overall cardiovascular risk among hypertensive patients (9). Recent advancements in genetic research have provided fresh insights into the intricate interplay between education and cardiovascular diseases. Mendelian randomization studies identified robust causal links between genetic variations related to education and specific cardiovascular conditions, such as coronary heart disease and heart failure (25, 26). However, to the best of our knowledge, our study represents the first comprehensive exploration of education in the context of CVDs employing MR methods.

Education exerts a significant influence on the development of CVDs, with various intermediate phenotypes contributing to this association. Previous studies have emphasized the predictive nature of education in terms of income, occupation, and health-related behaviors (27). Individuals with higher education levels tend to adopt healthier lifestyles compared to those with lower education levels (28). For instance, lower educational attainment has been causally linked to an increased risk of smoking (29), which subsequently contributes to unfavorable serum lipid levels, central obesity, higher resting heart rate, and poor fat distribution (30). Additionally, higher education levels have been associated with lower body mass index (BMI), suggesting a potential protective effect against obesity (31). The well-established relationship between BMI and CVD risk highlights the elevated risks of coronary heart disease, hypertension, and type 2 diabetes associated with increased BMI (32). Furthermore, studies have demonstrated that individuals with lower education levels have a significantly higher risk of coronary heart disease compared to those with higher education, with smoking and obesity identified as influential risk factors contributing to this disparity (33). Moreover, evidence suggests that the negative association between educational attainment and CVD incidence is mediated by factors such as diabetes, hypertension, and BMI (34). A recent Mendelian randomization study confirmed that the impact of education on CVD risk primarily operates through modifiable factors like BMI, systolic blood pressure, or smoking (35). It is crucial to consider potential confounding factors associated with education, including diverse socio-economic statuses and the cost of education. Higher socio-economic status generally correlates with better academic performance, healthier lifestyles, and improved access to healthcare, thereby influencing overall cardiovascular risk (36).

This study demonstrates several notable strengths. Firstly, the MR analysis conducted in this study yields compelling evidence supporting a causal link between education and CVDs. Secondly, the MR analysis effectively accounts for potential confounding factors and reverse causality, thereby providing accurate estimates of the causal effects. However, it is essential to acknowledge and address the limitations inherent in this study. The GWAS data employed primarily represent individuals of European descent, necessitating the validation of these findings within other ethnic groups to ensure their applicability outside of European populations. Furthermore, researchers must exercise caution when interpreting our study's outcomes. Education is influenced by a combination of social, environmental, and genetic factors, with genetic effects accounting for only approximately 20% of the variance. Consequently, genetic effects should not be construed as independent of environmental factors (37). Why education has such a small effect on peripheral arterial disease and hypertension, and why it has no effect on pulmonary embolism, still needs to be studied and explained in relation to a variety of other factors.

## 5 Conclusion

Our MR study yields compelling findings suggesting a causal relationship between higher educational attainment and a decreased risk of various CVDs, such as type 2 diabetes, coronary heart disease, ischemic stroke, cardiac-related mortality, heart failure, transient ischemic attack, atrial fibrillation, peripheral artery disease, and hypertension. However, the available evidence regarding the influence of education on pulmonary embolism remains limited. Consequently, it is imperative to acknowledge the significant impact of education in preventing CVDs, emphasizing the inclusion of education in public health initiatives and government policy-making, especially in educationally disadvantaged areas.

## Data Availability

The original contributions presented in the study are included in the article/Supplementary Material, further inquiries can be directed to the corresponding author.
